# Chylous ascites following radical nephrectomy: a case report

**DOI:** 10.1186/1752-1947-2-3

**Published:** 2008-01-11

**Authors:** Shahzad S Shah, Kamran Ahmed, Richard Smith, Ravi Mallina, Pouya Akhbari, Mohammad S Khan

**Affiliations:** 1 Department of Urology, Guy's Hospital, Guy's & St Thomas' NHS Foundation Trust & GKT School of Medicine, London SE1 9RT, UK

## Abstract

**Introduction:**

Chylous ascites may result from diverse pathologies. Ascites results either due to blockage of the lymphatics or leak secondary to inadvertent trauma during surgery.

**Case presentation:**

We report the first case of chylous ascites following radical nephrectomy for a renal cell carcinoma involving the right half of a crossed fused renal ectopia. The patient was managed conservatively.

**Conclusion:**

Post-operative chylous ascites is a rare complication of retroperitoneal and mediastinal surgery. Most cases resolve with conservative treatment which aims at decreasing lymph production and optimizing nutritional requirements along with palliative measures. Refractory cases need either open or laparoscopic ligation of the leaking lymphatic channels. A review of the current literature on the management of post-operative chylous ascites is presented.

## Introduction

Chylous ascites results from either blockage of the lymphatics or leakage secondary to inadvertent trauma during surgery. Most cases of traumatic chylous ascites resolve with conservative treatment but refractory cases may need surgical ligation of lymphatics. We report the first reported case of chylous ascites following radical nephrectomy for a renal cell carcinoma involving the right half of a crossed fused renal ectopia. The chylous ascites resolved with conservative management. A brief review of the literature on the management of post-operative chylous ascites is presented.

## Case presentation

**Figure 1:**
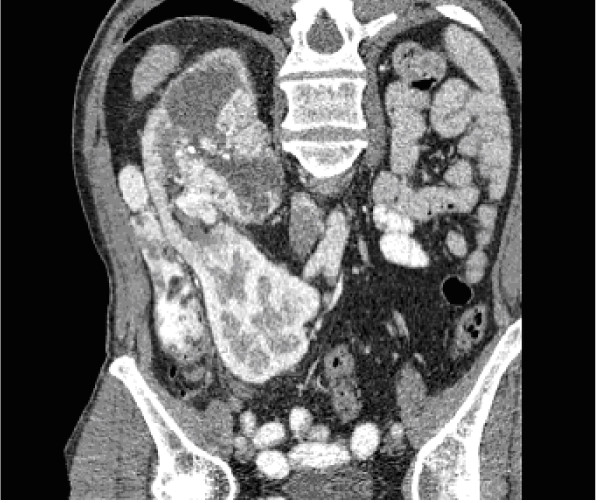
CT scan showing tumour in the upper moiety of the crossed fused renal ectopia.

A 60-year old male presented with acute right loin pain and frank haematuria. He was hypertensive but well controlled on medication. He had undergone coronary artery bypass grafting 9 years earlier. Physical examination was normal apart from a median sternotomy scar. Urine was sterile on culture and showed no malignant cells on cytology. Urea, creatinine and electrolytes were within normal range. Ultrasound scan showed no kidney in the left renal area and a 7 × 5 × 5 cm heterogenous irregular mass arising from the mid-pole of the right kidney. CT scan confirmed the presence of a large complex mass measuring 11.6 × 8 × 6.5 cm arising from the mid and upper pole of the right kidney. In addition it showed a cross fused left kidney in the right iliac fossa (Fig. 1). There was a single aorto-caval lymph node measuring 8 mm but no pulmonary metastases.

An open right radical nephrectomy was performed. The dissection of the kidney was straightforward. The isthmus between the right and left kidney was transected without any complications and the raw surface of the left kidney over-sewn with surgical bolsters. A para-aortic lymph node dissection was undertaken between the superior mesenteric artery and bifurcation of the aorta. The surgical procedure did not differ from a standard radical nephrectomy except for the division of the isthmus. On histology, the tumour was a classical clear cell adeno-carcinoma with no nodal metastases (pT2N0).

**Figure 2:**
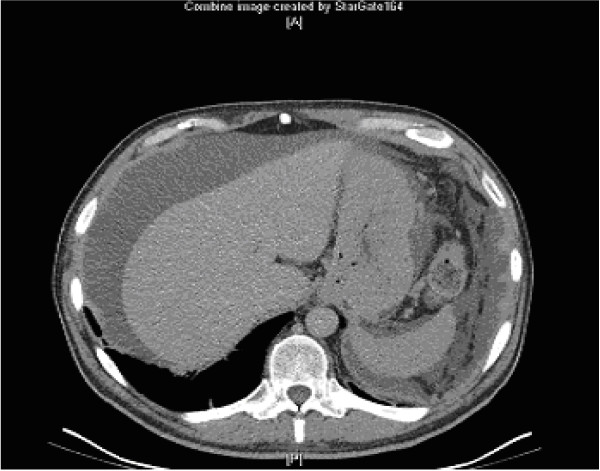
Post-operative chylous ascites.

Post-operatively the patient had copious (150–200 mls daily) drainage via a retroperitoneal drain which on biochemical analysis was consistent with serum. Hence the drain was removed. The patient was discharged on day 5 but was readmitted three weeks later with abdominal distension and pain. Clinically he had ascites. This was confirmed on CT scan (Fig. 2). Paracentesis and biochemical analysis were consistent with chylous ascites.

**Figure 3:**
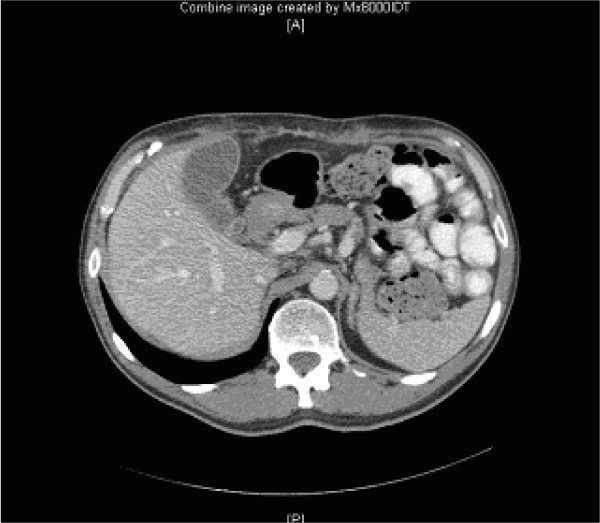
Resolution of ascites following conservative treatment.

The patient was initially managed with oral diuretics (Furosemide 40 mg twice daily & Spironolactone 25 mg 8 hourly). Treatment resulted in hyponatraemia and hypotension without any improvement in ascites and hence was discontinued. A therapeutic paracentesis was performed to alleviate abdominal discomfort. The patient was then commenced on parenteral nutrition and medium chain triglycerides. This resulted in gradual resolution of ascites and no reaccumulation during two months of follow up (Fig. 3).

## Discussion

Chylous ascites is a rare condition. Its etiological factors can be broadly classified as congenital, infective, neoplastic and traumatic or post surgical. The majority of cases are caused by diseases that interfere with abdominal or retroperitoneal lymphatic drainage. Amongst surgical procedures, vascular operations account for the majority of post-operative chylous ascites [1]. This complication may become evident within a few days following surgery or take several months [2].

The lymphatic drainage from the kidney and testes is to the retroperitoneal para-aortic nodes. Thus chylous ascites is a well recognized complication of retroperitoneal node dissection (RPLND) for testicular cancer. However, only 34 cases of chylous ascites have been reported in the English medical literature following renal surgery for diverse indications. These included twelve (n = 12) after radical nephrectomy for Wilm's tumour, nine (n = 9) for renal cell carcinoma, eight (n = 8) after laparoscopic donor nephrectomy, two (n = 2) following nephro-ureterectomy and one each for renal abscess, renal trauma and non-functioning kidney.

Presentation of post-operative chylous ascites is similar to ascites due to other causes including progressive abdominal distention and weight gain. The patient may complain of dyspnoea due to reduced diaphragmatic movements or chylothorax. Non-specific symptoms include nausea, vomiting or post operative wound leakage. The diagnosis can be confirmed by abdominal paracentesis. The aspirate is typically milky white and stains positive for fat with Sudan III. Its specific gravity is greater than 1.012 and has alkaline pH. Cytology shows predominantly lymphocytes. Chemical analysis reveals high triglyceride levels 2–8 fold that of plasma (range 0.4–4 gm/dl) and protein content greater than 3 gm/dl. Serum abnormalities include hypoalbuminaemia, lymphocytopenia and anemia secondary to protein loss and malnutrition. Occasionally the diagnosis is evident only on exploration.

Bipedal lymphangiography with ethiodized oil injected into lymphatic vessels on the dorsum of the foot has been the traditional way of mapping the lymphatic tree. Lymphangiography however, is technically challenging, time consuming and has the additional disadvantage of staining the operative field. Therefore lymphangiography has been abandoned in favour of newer radiological techniques [3].

Pui and Yueh report their experience with ^99m^technetium (Tc)-antimony sulfide colloid, human albumin or dextran-scintigraphy for chylous collections. They claim that lymphoscintigraphy can accurately pin point lymphatic leaks and thus may be a useful tool in selecting patients for surgery [4].

CT scan remains the imaging of choice. It may point to the diagnosis by demonstrating simultaneous intraperitoneal and extraperitoneal fluid collections following retroperitoneal nephrectomy. As the density of the chylous fluid is identical to water, it is indistinguishable from clear ascites, urine or bowel fluid but the pathognomonic feature of chylous ascites on CT scan is," fat fluid level", which may be demonstrable, if the patient is imaged after a prolonged period of lying supine [5].

The treatment for chylous ascites is aimed at alleviation of the discomfort associated with the distended abdomen, reducing the flow of lymph to the mesenteric lymph nodes, which join together to form the retroperitoneal lymph channels, and replacement of the nutritional losses.

These objectives are achieved through therapeutic paracentesis, when required, in combination with diuretics and restricted salt intake, a high protein, low fat, medium chain triglyceride diet, and parenteral nutrition [3]. Somatostatin has recently been shown to be effective in the treatment of this condition [6].

Paracentesis may be performed early both as a diagnostic tool and as a palliative measure. Its main advantage is its immediate palliation but paracentesis is not effective on its own unless combined with other conservative measures. Repeated drainage of the ascitic fluid may prolong the leak, depress immunity and increase nutritional requirements [2].

Dietary intervention remains the mainstay of conservative treatment of chylous ascites and consists of a high protein, low fat, medium chain triglyceride diet. The rationale for using medium chain triglycerides is the fact that these bypass the lymphatic channels of the gut and enter directly into the portal venous system in contrast to long chain triglycerides which enter the portal venous blood through the lymphatics of the bowel. It has been recommended that medium chain triglycerides should be continued for several months after resolution of the ascites [3].

Total parenteral nutrition is an essential component in the management of chylous ascites and serves two important objectives. It fulfills the nutritional requirement of these patients, most of whom are malnourished due to their inability to tolerate oral feeding. More importantly it helps to decrease the production of lymph and allows the bowel to rest. The resolution of chylous ascites is reported to occur within 2–6 weeks in 60–100% patients with TPN alone, or in combination with medium chain triglycerides and paracentesis [7]. Although not all patients respond to TPN alone this should be part of any conservative treatment plan.

Somatostatin is a naturally occurring peptide consisting of 14 to 28 amino acids. It is found in the central nervous system, gastrointestinal tract and the pancreas. It decreases the intestinal absorption of fats, lowers triglyceride concentration in the thoracic duct and attenuates the lymph flow in the major lymph vessels. It also decreases splanchnic blood flow. Analogues now available are octreotide and lanreotide which are octapeptides with a much longer half life than somatostatin. Since somatostatin interferes with blood glucose regulation, close monitoring of blood glucose is recommended during its administration [8].

Other therapeutic measures include intravenous etilefrine, a sympathomimetic drug which acts by contracting the smooth muscle of the thoracic duct thereby decreasing the flow of chyle.

Surgical intervention is needed if the lymphatic leak persists in spite of maximal conservative therapy for several weeks. This usually entails either direct suture ligation of the disrupted lymphatic channels or insertion of a peritoneovenous shunt [1].

If surgical intervention becomes mandatory, in some cases the site of the fistula may be visible. Identification of the fistula may be helped by a fatty meal taken pre-operatively or by intra-operative injection of a contrast. Suture ligation of the lymphatics results in termination of the leak. If a definitive leak site can not be identified, suturing of the retro-aortic tissues en-mass may resolve the lymphatic leak [2]. Better outcomes of surgery are expected in patients with accurate localization of the leak. The main disadvantage of surgery is the hazard of re-operating on already compromised patients who are just recovering from major surgical trauma. Despite its disadvantages surgical therapy remains an effective option for refractory cases [9].

There is no consensus as regards the exact timing of operative intervention but it is generally recommended that conservative therapy should be tried for at least 4–8 weeks [1].

Peritoneovenous shunting is an alternative to exploration in patients with rapid accumulation of ascitic fluid. This avoids nutritional depletion as the fluid is re-circulated. Shunts are associated with fewer complications than repeat paracentesis but complications like disseminated intravascular coagulation (DIC), fat embolism and fatal sepsis may occur [3].

Cope describes a technique of catheterization of the cysterna chyli and major retroperitoneal lymphatic ducts by percutaneous transabdominal puncture and embolisation of the leaking lymphatic trunk but the safety of this technique is yet to be established [10].

The prognosis of patients with chylous ascites depends upon the condition causing the leak, associated co-morbidities and the pathological condition for which surgery was performed in the first place. Generally the prognosis of patients with non-surgical ascites is poorer because of underlying causes. Post-operative chylous ascites has a better prognosis. Pabst et al, in a review of 17 published reports, documented successful resolution of post-operative chylous ascites in 92.3% of patients, the majority of patients responding to conservative therapy [2].

## Conclusion

Post-operative chylous ascites is a rare complication of retroperitoneal and mediastinal surgery. The condition poses a difficult management problem. Most cases resolve with conservative treatment which usually involves a prolonged period of multimodal therapy aimed at decreasing lymph production and optimizing nutritional requirements along with other palliative measures. Refractory cases may need either open or laparoscopic ligation of the leaking lymphatic channels.

## Competing interests

The author(s) declare that they have no competing interests.

## Authors' contributions

All authors have read and approved the final manuscript.

SSS acquired patient records, data and drafted the manuscript.

KA participated in acquisition of data.

RS participated in acquisition of data.

RM participated in acquisition of data.

PA participated in acquisition of data.

MSK carried out the design of the study, coordinated the study, drafted the manuscript and obtained consent from the patient.

## Consent

"Written informed consent was obtained from the patient for publication of this case report and any accompanying images. A copy of the written consent is available for review by the Editor-in-Chief of this journal."
